# Protective and Therapeutic Effects of Chinese Medicine Formula Jiajian Yunvjian on Experimental Cardiac Remodeling after Myocardial Infarction Induced by Coronary Artery Ligation

**DOI:** 10.1155/2015/949656

**Published:** 2015-06-22

**Authors:** Jun Du, Wei-liang Gu, Chang-xun Chen, Ying Wang, Jian Lv

**Affiliations:** Department of Pharmacology, Shanghai University of Traditional Chinese Medicine, Shanghai 201203, China

## Abstract

*Introduction*. This study was designed to explore the effect and mechanism of a classic Chinese medicine formula Jiajian Yunvjian (JJYNJ) on cardiac remodeling. Cardiac remodeling after myocardial infarction (MI) model was achieved by coronary artery ligation (CAL). *Methodology*. When dosed orally once daily, the effects of JJYNJ on hemodynamics, left ventricular weight index (LVWI), heart weight index (HWI), concentration, and gene expression of neuroendocrine factors as well as the histomorphological observation were determined. *Results*. After 4 weeks, mild cardiac remodeling in CAL group was characterized compared with sham group, but after 4 weeks of treatment of JJYNJ, hemodynamics improved, HWI reduced, and circulating angiotensin II (Ang II), endothelin-1 (ET-1), tumor necrosis factor-*α* (TNF-*α*), and hydroxyproline (Hyp) concentrations as well as Ang II receptor type 1 (AT_1_R) mRNA, transforming growth factor *β*_1_ (TGF-*β*_1_) mRNA, and TNF-*α* mRNA levels in myocardium were lower than in CAL group. Decreased plasma aldosterone (ALD) concentration, cross-sectional area of cardiomyocyte, collagen volume fraction (CVF), collagen types I and III, perivascular collagen area (PVCA), and upregulated nitric oxide (NO) levels were observed at the same time. *Conclusions*. These findings suggest that JJYNJ may have a protective and therapeutic function on cardiac remodeling related to MI.

## 1. Introduction

Cardiac remodeling is an important and fundamental adaptive response to a requirement for increased contractile power. It is not only a factor intimately associated with chronic heart failure (CHF), but also an important cause of increased morbidity and mortality of cardiac diseases [1]. Hence, it is regarded as a major risk factor of heart disease. A growing body of experimental evidence shows that the rennin-angiotensin-aldosterone system (RAAS) and sympathetic nervous system (SNS) play pivotal effects in the progress of cardiac hypertrophy [2, 3].

JJYNJ is a traditional Chinese medicine (TCM) formula from a classic literature (*Wenbing Tiaobian*) used for more than two hundred years. JJYNJ contains 5 mineral and herbal medicines:* gypsum* (CaSO_4_·2H_2_O), rhizome of* Anemarrhena asphodeloides* Bge., root of* Scrophularia ningpoensis* Hemsl., root of* Rehmannia glutinosa* Libosch, and root tuber of* Ophiopogon japonicus* (L.f.) KER-Gawl. In TCM theory, JJYNJ can nourish yin to lessen fire, which increases a patient's ability to keep the balance of yin and yang and protect from invasion by internal or external pathogenic influences. In China, some Chinese physicians succeeded by using JJYNJ in the treatment of infectious and febrile diseases, such as measles, and epidemic hemorrhagic fever.

Some reports have revealed that gypsum has significant antipyretic effect and can decrease vascular permeability, acting as an anti-inflammatory agent [4].* Anemarrhena* rhizome has an inhibiting effect on proliferation and protective role in ischemia induced brain injury as well [5, 6].* Scrophularia* root has a beneficial effect on ventricular remodeling [7, 8].* Rehmannia* root can inhibit blood platelet aggregation [9] and has antioxidant and anti-inflammatory effects [10, 11].* Ophiopogon* root can enhance endothelial cell protective and antiadhesive activities [12] and antithrombotic activity [13]. However, if these herbs were used singly, it would be insufficient to protect the body from the pathogens and mediate the balance of yin and yang, but a combination of these 5 herbs can strengthen the body to protect from internal and external pathogens. Since MI can be considered one of the pathogens resulting in cardiac remodeling, we deduced that JJYNJ would have some beneficial effects on cardiac remodeling.

In the present project, we examined the pharmacological effects of JJYNJ on cardiac remodeling induced by coronary artery ligation (CAL) and the underlying mechanism of its action was revealed to a certain extent. In our laboratory, we have confirmed JJYNJ has some activities in downregulating SNS excitability. In order to evaluate its antiremodeling effects, which may be one of the potential characteristics of TCM, we designed experimental protocols that can exhibit JJYNJ's protective and curative effects on this disease.

## 2. Materials and Methods

### 2.1. Animals

Adult male Sprague-Dawley rats (weighting 200–220 g) from laboratories of Academia Sinica were housed in a 12 : 12 h light-dark cycle room with the temperature of 23 ± 1°C and given standard chow and tap water. They were housed for at least 3 days before the start of the experiment. This study was approved by the Animal Ethics Committee of Shanghai University of Traditional Chinese Medicine.

### 2.2. Coronary Artery Ligation

Cardiac remodeling was induced by ligating the left anterior descending coronary artery in rats as previously described [14]. Briefly, rats were anesthetized with sodium pentobarbital (35 mg·kg^−1^, i.p.), intubated, and artificially ventilated with air. The left anterior descending coronary artery was ligated with a 6-0 silk suture approximately 2 mm from its origin. Ligation was deemed successful when the anterior wall of the left ventricle (LV) turned pale and ischaemia was confirmed by the raising of ST recorded by an electrocardiogram. Meanwhile, sham operation as a parallel control was identical except that the ligature was placed and then removed without tightening it. Rats were kept in a heating pad and monitored until awake. The surgical procedure resulted in 15% mortality. For evaluation of the effects of long term treatment with JJYNJ, rats with surgery were randomly divided into the two groups: CAL group (*n* = 10), JJYNJ group (*n* = 10). The treatment started from the next day after surgery. JJYNJ group was treated with JJYNJ water extract 20 mL·kg^−1^ (equal to crude drug 16.2 g·kg^−1^) by gavage once daily, while the CAL group and sham operated group (*n* = 8) were both orally treated with distilled water once daily.

### 2.3. Hemodynamic Parameter Measurements In Vivo

Four weeks after the operation, heart rate (HR), systolic blood pressure (SBP), diastolic blood pressure (DBP), mean arterial pressure (MAP), left ventricular systolic pressure (LVSP), left ventricular end diastolic pressure (LVEDP), +LV *dp*/*dt*_max_, and −LV *dp*/*dt*_max_ of sham, CAL, and JJYNJ animals were measured. The animals were weighed and anesthetized by sodium pentobarbital (35 mg·kg^−1^, i.p.), and then left and right carotid arteries were separated and cannulated with a 24-gauge polyethylene catheter filled with heparin/saline solution to record the blood pressure [15]. The transducer (Baxter, model MP5100) was connected to a Med-laboratory system (model 400/s, AD Instruments), after the blood pressure was measured; then the catheter was advanced into the left ventricle to record left ventricular pressure and function. Electrocardiogram was also used to record heart rate at the same time.

### 2.4. Ratio of HW and LVW to BW

After measurement of hemodynamic parameters, the body weight (BW) was measured and then the blood was collected for plasma analysis. The heart was isolated; heart weight (HW), left ventricular weight (LVW), heart weight index (HWI, HW/BW), and left ventricular weight index (LVWI, LVW/BW) were determined for each animal. Left ventricle was quickly dissected and sectioned into two parts. The upper part was immediately transferred into liquid nitrogen and then stored at −80°C for biochemical and gene analysis. The lower part was stored in 10% formaldehyde to prepare paraffin section for histopathology.

### 2.5. Measurement of Collagen Content

For detecting the collagen synthesis in the myocardium, about 200 mg left ventricular tissue was weighted by precision electronic balance and chromatometry using an ultraviolet spectrophotometer was applied to measure the concentration of collagen and the hydroxyproline (Hyp) concentration 4 weeks after surgery. The LV tissue protein was measured at the same time.

### 2.6. Measurement of Neuroendocrine Factors

To assess the inhibitory potency of JJYNJ on cardiac remodeling, we measured the neuroendocrine factors: angiotensin II (Ang II), aldosterone (ALD), endothelin-1 (ET-1), nitric oxide (NO), and tumor necrosis factor-*α* (TNF-*α*) activity in the myocardium of sham, CAL, and JJYNJ group rats. For the measurement of Ang II, ALD, ET-1, and TNF-*α* activity, we employed radioimmunoassay. Chromatometry using an ultraviolet spectrophotometer was applied to measure the activity and concentration of NO and LV tissue protein. Nitrate reductase was used as a reductant for NO based on its specificity [16].

### 2.7. Morphometric Measurement

For histological analysis of cell size and cardiac fibrosis in terms of LV myocardial fibrillar collagen composition and distribution, hearts were fixed with 10% formaldehyde and embedded in paraffin, sectioned at 4 *μ*m thickness, and stained by hematoxylin and eosin (HE) and picric Sirius red methods for cardiomyocyte and collagen measurements. To determine the degrees of cross-sectional area of cardiomyocyte and collagen fiber accumulation, we selected 5 fields randomly and chose 30 myocardial cells per field to calculate cell size and selected another 5 fields randomly and calculated the ratio of picric Sirius red stained fibrosis area to total myocardial area with the image analysis software image-pro analysis 5.0. The perivascular fibrosis was determined as the ratio of the area of fibrosis surrounding the vessel wall to the total vessel area [17]. The contents of subtype I and III collagen in myocardium were determined using microscope (IX70, Olympus, Japan) with polarimetric filter analysis at the same time.

### 2.8. Analysis of AT_1_R, TGF-*β*_1_, and TNF-*α* mRNA by Real Time Reverse Transcription Polymerase Chain Reaction (RT-PCR)

Quantitative RT-PCR was performed in the iCycler iQ (Bio-Rad, CA) using SYBR Green I dye (Qiagen, CA), as described by the manufacturer. Each reaction volume for preparation of cDNA was 20 *μ*L containing 4 *μ*L of RNA, 4 *μ*L of buffer (5x), 1 *μ*L of primers (Quantitect Primer Assays, Qiagen), 0.5 *μ*L of dNTPs, 0.5 *μ*L of oligo (dT), 1 *μ*L of reverse transcriptase, and 10 *μ*L of DEPC H_2_O. Each reaction volume for mRNA was 50 *μ*L and contained 2 *μ*L of cDNA, 1 *μ*L of sense and antisense primers, 32.5 *μ*L of SYBR Green PCR master mix, and 14.5 *μ*L of ddH_2_O. A typical protocol included reverse transcription at 37°C for 60 min and a denaturation step at 95°C for 10 min followed by 40 cycles with 95°C denaturation for 20 s, 55°C annealing for 30 s, and 72°C extension for 30 s. The sequences of primer were as follows: TGF*β*_1_ mRNA sense: 5′-GCTGCTGACCCCCACTGAT-3′, TGF*β*_1_ mRNA antisense: 5′-TGCCGGACAACTCCAGTGA-3′; AT_1_R mRNA sense: 5′-GCACACTGGCAATGTAATGC-3′, AT_1_R mRNA antisense: 5′-GTTGAACAGAACAAGTGACC-3′; TNF-*α* mRNA sense: 5′-TGACTTTCTCCTGGTATGAAATGG-3′, TNF-*α* mRNA antisense: 5′-TGACTTTCTCCTGGTATGAAATGG-3′; GAPDH sense: 5′-CCGAGGGCCCACTAAAGG-3′, GAPDH antisense: 5′-GCTGTTGAAGTCACAGGAGACAA-3′. Detection of the fluorescent product was performed at the end of the extension period. To confirm amplification specificity, the PCR products were subjected to a melting curve analysis. Negative controls containing water instead of RNA were concomitantly run to confirm that the samples were not cross contaminated. Real time PCR data were analyzed with a standard curve. Correlation coefficients for the standard curves all were >0.90. Targets were normalized to reactions performed using Quantitect GAPDH primers (Qiagen), and fold-change was determined using the comparative threshold method [18].

### 2.9. Statistical Analysis

All data are presented as the means ± standard deviation. Statistical analysis was performed by repeated one-way analysis of variance (ANOVA), combined with Dunnett's multiplex comparison analysis. A probability value of less than 0.05 was considered to be statistically significant.

## 3. Results

### 3.1. Changes in Hemodynamic Parameters

Changes in hemodynamic parameter after 4 weeks of CAL surgery are listed in Table 1. The values of SBP, DBP, PP, LVSP, and −*dp*/*dt*_max_ were markedly (*p* < 0.05) lowered in CAL group compared to the matched sham group, but no changes were showed in HR, MABP, +*dp*/*dt*_max_, and *t* − *dp*/*dt*_max_ between the two groups. But LVEDP, a figure of heart failure, was significantly (*p* < 0.05) elevated after surgery. JJYNJ could markedly (*p* < 0.05) improve SBP level and reduce LVEDP level throughout the experimental period, but there were no significant differences in PP, LVSP, +*dp*/*dt*_max_, and −*dp*/*dt*_max_ level between CAL and JJYNJ treated CAL groups.

### 3.2. Changes in HW, LVW, BW, LVW/BW, and HW/BW

Changes in HW, LVW, BW, LVW/BW, and HW/BW after 4 weeks of CAL surgery are listed in Table 2. Ratios of LVW/BW and HW/BW in the CAL group were increased significantly (*p* < 0.05), compared to control animals of the sham group. There are no changes in LVW, HW, and BW between the two groups. JJYNJ could significantly (*p* < 0.05) decrease HW/BW after 4 weeks of CAL, but there were no significant changes in LVW and HW except a slight lower ratio of LVW/BW when compared with the same time period in the CAL group and there was a reversed increase (*p* < 0.05) of BW in the JJYNJ treated CAL group.

### 3.3. Changes in Myocardium Collagen Content

**Figure 1:**
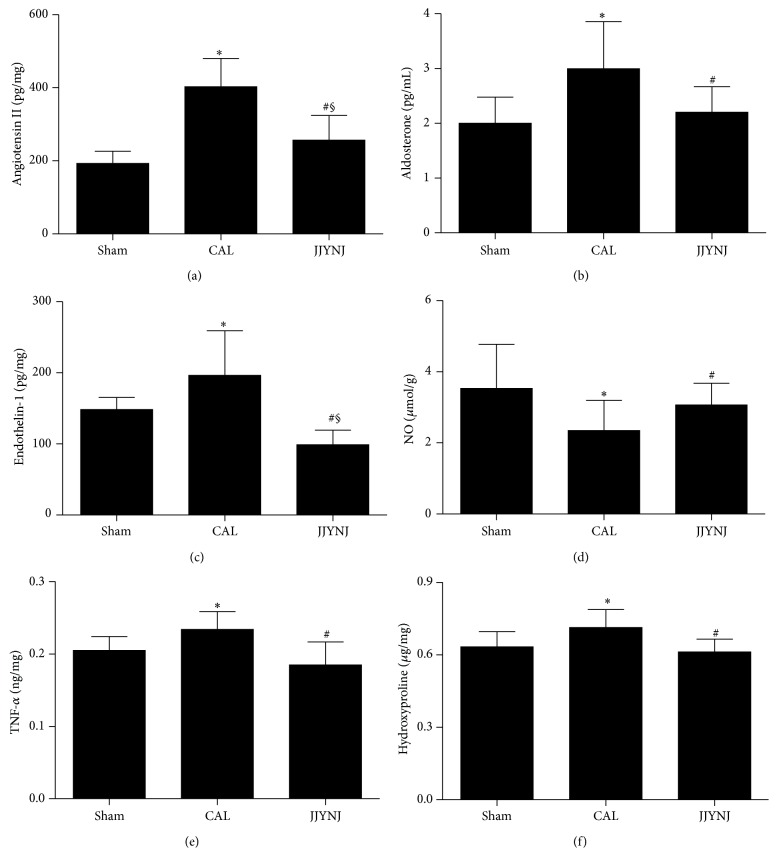
Comparison of myocardium Ang II (a), ALD (b), ET-1 (c), NO (d), TNF-*α* (e) and Hyp (f) levels 4 weeks after coronary artery ligation in sham, CAL and JJYNJ groups. Each bar is expressed as means ± standard deviation, ^*∗*^ *p* < 0.05 when compared to the sham; ^#^ *p* < 0.05 when compared to the CAL; ^§^ *p* < 0.05 when compared to the sham.

The content of Hyp in LV tissue was significantly (*p* < 0.05) increased in CAL group rats 4 weeks after surgery, compared to the same time period for the sham group, which indicated the onset of LV hypertrophy and fibrosis induced by CAL. But it was significantly (*p* < 0.05) decreased in the JJYNJ treated CAL group, compared with the same time period in the CAL group (Figure 1(f)).

### 3.4. Cardiac Hypertrophic Neuroendocrine Factor Expression

The changes in level of cardiac hypertrophic marker (Ang II, ALD, ET-1, NO, and TNF-*α*) expression 4 weeks after surgery are shown in Figure 1. The levels of Ang II, ALD, ET-1, and TNF-*α* were markedly (*p* < 0.05) elevated in the left ventricle of the CAL group, compared to those of the same time period of sham group. However, the level of NO was significantly (*p* < 0.05) lower in the CAL group than that in sham group. But the concentrations of Ang II, ALD, ET-1, and TNF-*α* were markedly (*p* < 0.05) decreased in the left ventricle of the JJYNJ treated CAL group, compared to that of the CAL group (Figures 1(a)–1(c) and 1(e)). A significant (*p* < 0.05) increase in the concentration of NO was observed in the JJYNJ treated CAL group, compared to the same time period in the CAL group (Figure 1(d)).

### 3.5. Changes in Morphometric Measurement

**Figure 2:**
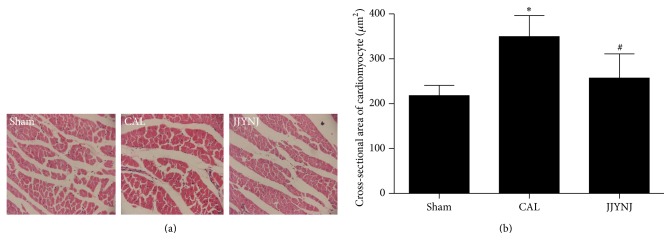
Hematoxylin and eosin staining demonstrating cross-sectional area of cardiomyocyte of left ventricular tissue section from sham, CAL, and JJYNJ treated rats 4 weeks after coronary artery ligation. Each bar is expressed as means ± standard deviation; ^*∗*^ *p* < 0.05 when compared to the sham; ^#^ *p* < 0.05 when compared to the CAL (magnification: ×400).

**Figure 3:**
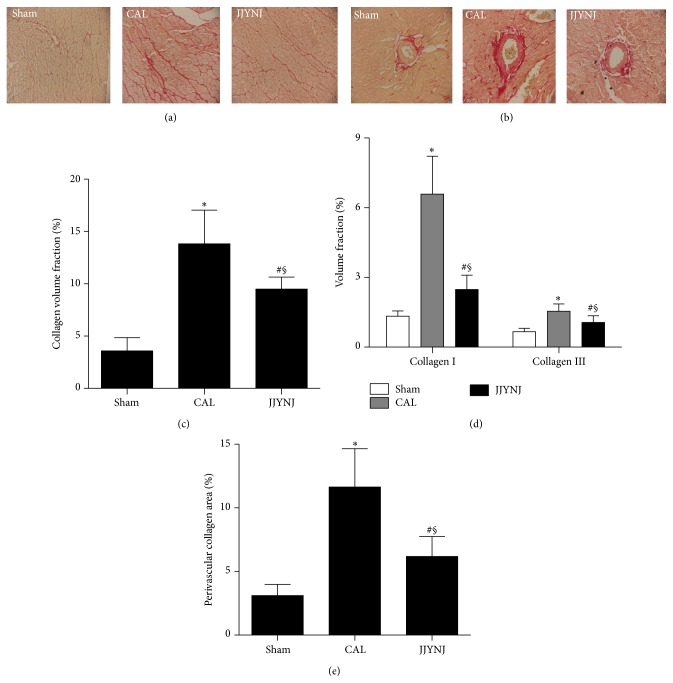
Representative photomicrographs (a and b) of left ventricular tissue section stained by picric Sirius red obtained from sham, CAL, and JJYNJ treated rats 4 weeks after coronary artery ligation. Relative quantitative analysis was performed for CVF (c), collagen I and collagen III (d), and PCVA (e). Each bar is expressed as means ± standard deviation; ^*∗*^ *p* < 0.05 when compared to the sham; ^#^ *p* < 0.05 when compared to the CAL; ^§^ *p* < 0.05 when compared to the sham (magnification: ×400).

The average cross-sectional area of cardiomyocyte was markedly (*p* < 0.05) increased in the CAL group 4 weeks after surgery, compared to that of the sham group (Figures 2(a)-2(b)). However, the average cardiomyocyte cross-sectional area was significantly (*p* < 0.05) decreased in JJYNJ treated CAL group, compared to the same time period of the CAL group. The percentage of cardiac fibrosis was markedly (*p* < 0.05) increased in both CAL and JJYNJ treated CAL groups, compared to that of the sham group (Figures 3(a)–3(e)). However, the percentage of cardiac fibrosis was significantly (*p* < 0.05) decreased in JJYNJ treated CAL group compared to that in the CAL group.

**Figure 4:**
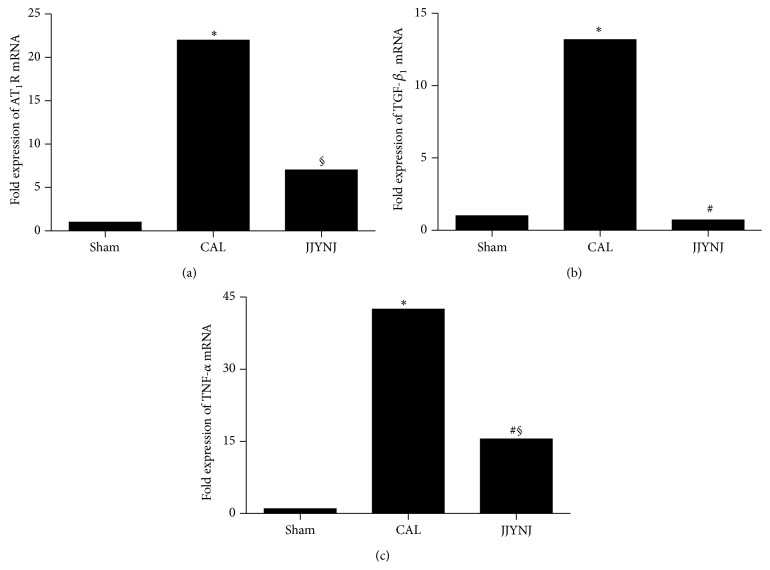
Real time RT-PCR was performed for analyzing AT_1_R mRNA (a), TGF*β*_1_ mRNA (b), and TNF-*α* mRNA (c) expression using left ventricular tissue obtained from sham, CAL, and JJYNJ treated rats 4 weeks after coronary artery ligation. GAPDH was used as a quantitative control. Each bar is expressed as means; ^*∗*^ *p* < 0.05 when compared to the sham; ^#^ *p* < 0.05 when compared to the CAL; ^§^ *p* < 0.05 when compared to the sham.

Figure 4 shows that AT_1_R, TGF-*β*_1_, and TNF-*α* mRNA expression levels in left ventricular tissue increased significantly (*p* < 0.05) 4 weeks after MI in the CAL group analyzed by real time RT-PCR. The sham operated group displayed low level expression of AT_1_R, TGF-*β*_1_, and TNF-*α* mRNA in left ventricular tissue. The JJYNJ treated CAL group also showed a diminishment (*p* < 0.05) of TGF-*β*_1_ and TNF-*α* mRNA transcription; AT_1_R mRNA expression in left ventricular tissue was decreased 4 weeks after myocardial infarction but showed no obvious changes.

## 4. Discussion and Conclusions

In the early period, the heart undergoes adaptive responses to the changes in hemodynamics as less myocardium attempts to maintain the same cardiac output as before. Increased levels of some neuroendocrine factors may be a beneficial “adaptation” initially, but over time, they become deleterious, leading “maladaptation” eventually to CHF [19]. RAAS is the major system that has enhanced activity after MI. When the body responds to the diminished cardiac output, Ang II is markedly elevated and ALD is released. All of these effects serve to increase plasma volume, systemic arteriolar constriction, endothelial dysfunction, and vascular and sympathetic tone, decrease sodium excretion, and induce left ventricular remodeling [20–22]. Ang II is the main effector of RAAS and causes myocardial and vascular hypertrophy, increases collagen synthesis, and inhibits collagenase activity in cardiac fibroblasts [23]; Ang II and AT_1_R are upregulated in the heart after MI; most of physiological effects of Ang II are meditated by AT_1_R [24]. Within the region of the myocardial infarct, ALD increases the deposition of collagen, myocardial electrical instability, and deaths of cardiomyocytes and its production is mediated by Ang II via AT_1_R on cardiomyocytes [25]. Data has also shown elevation of TNF-*α* that is associated with the marked activation of the RAAS and stimulation of pathophysiological concentration of TNF-*α* provokes a time dependent increase in LV remodeling in animal models of HF [26]. Via activation of TNF-*α*, the sympathetic nervous system has been implicated in inducing apoptosis in cardiomyocytes [27]. Treatment with TNF-*α* could upregulate expression of AT_1_R on cardiac fibroblasts [28]. The further loss of cardiomyocyte contributes to progressive LV dilatation. The RAAS and SNS systems play an important role in cardiac remodeling after MI.

JJYNJ is used clinically to treat febrile disease. In this study, we used animal experiments to evaluate the effectiveness of this formula in cardiac remodeling according to the characteristics of TCM, which works by improving the animal's whole body and neuroendocrine action. Our data showed that JJYNJ significantly increased SBP, LVSP and decreased DBP, LVEDP, demonstrating that it could improve cardiac function to some extent. The degree of cardiac hypertrophy was assessed by the increase of LVWI, HWI, and the cardiomyocyte cross-sectional area. Administration of JJYNJ for 4 weeks prevented cardiac hypertrophy. Additionally, JJYNJ could decrease Ang II, ALD, and Hyp contents and downregulate the expression of AT_1_R and TNF-*α* mRNA, which significantly attenuate the experimental cardiac remodeling; the mechanism was probably related to its ability to inhibit activation of RAAS.

TGF-*β*_1_ signaling is crucial for repression of inflammatory gene synthesis in postinfarction healing, mediating resolution of inflammatory infiltration and is a key mediator in the pathogenesis of hypertrophic and dilative ventricular remodeling by stimulating cardiomyocyte growth and inducing interstitial fibrosis. TGF-*β*_1_ modulates proliferation and conversion fibroblast phenotype and gene expression, promotes extracellular matrix (ECM) deposition in the infarct by upregulating collagen and fibronectin synthesis and hypertrophic growth of cardiomyocytes, and decreases matrix degradation [29, 30]. Though activation of TGF-*β*_1_ may be a protective effect against ischemic myocardial damage during the early phase, inhibition of TGF-*β*_1_ signaling exacerbates early cardiac dysfunction but prevents late remodeling after infarction [31]. TGF-*β*_1_ and Ang II are connected in the pathogenesis of cardiac remodeling. Ang II could upregulate TGF-*β*_1_ expression via activation of AT_1_R in cardiomyocyte and fibroblast [32]. TGF-*β*_1_ also can mediate Ang II induced structural ventricular remodeling in an auto-/paracrine manner [33, 34].

ET-1 is a potent vasoconstrictor and mitogenic peptide via its interaction with a special receptor in the heart, though local ET-1 is necessary to maintain normal cardiac function and cardiomyocyte survival by modulating TNF-*α* related apoptosis, in part through upregulation of NF-*κ*B signaling [35, 36]. In addition to its vasoconstrictor actions, ET-1 plays a critical role in the functional deterioration of the left ventricle during the transition from compensatory hypertrophy to CHF [37]; it could stimulate release of renin and ALD, promote the conversion of Ang I to Ang II in endothelial cells, stimulate the proliferation of vascular smooth muscle cells and fibroblast, and increase certain protooncogene expression [38].

NO activates soluble guanylyl cyclase, which in turn produces cGMP and results in decreased intracellular Ca^2+^ and vasorelaxation of smooth muscles [39, 40]. It is reported to have antihypertrophic effects in vitro in several cell types including cardiomyocytes. Thus, it would be expected that chronic inhibition of NO synthesis induces cardiac hypertrophy in vivo. NO also significantly inhibited ET-1 stimulated promoter activity of hypertrophic marker gene *β*-MHC and the enhanced protein synthesis and NO is a negative regulator in ET-1 induced cardiac hypertrophy [41]. However, the role of NO in cardiac hypertrophy is still unclear because it is difficult to assess the growth inhibitory effect of NO independently of changes in cardiac loading conditions [42].

The present study demonstrated that ET-1, NO, TNF-*α*, and TNF-*α* mRNA expression increased significantly following CAL. But treatment with JJYNJ decreased these contents and lowered the expression of TGF-*β*_1_ mRNA.

Furthermore, JJYNJ's inhibitory effects on cardiac remodeling were also associated with reduction of cardiac fibrosis in left ventricle. In pathological remodeling, the collagen concentration rises disproportionately, which both contributes to tissue stiffness and promotes cardiomyocyte atrophy [43]. Therefore, the suppressive effect of JJYNJ on collagen may provide a beneficial effect on left ventricular structure and function.

Although acute diseases can be treated and mortality from disease has declined in industrialized countries as a result of progress in modern medical treatment, most modern allopathic medicines cannot cure chronic diseases such as hypertension, diabetes, and allergic diseases [44]. These drugs usually contain only one active compound with sharply focused pharmacological activity, and the compounds sometimes cause unfavorable side effects. Based on these factors, many patients choose to explore complementary and alternative medicines, so TCM has attracted more attention for its potency to treat patients holistically. In this study, we revealed that JJYNJ, one of the herbal medicinal formulas in TCM, has holistic effects on cardiac remodeling, that is, preventive and fundamentally curative effects. There are few articles in the pharmacological literature mentioning that medicinal treatments of this sort show some therapeutic effects, and we hope this study will contribute to the research evaluating experimental evidences of the result of using traditional medicine. This study supplies experimental evidences that JJYNJ would be effective against cardiac remodeling. Further studies are needed to evaluate the action mechanisms of JJYNJ as well as the pharmacological effects of each medicinal herb in modern science.

In summary, our results suggest that JJYNJ has protective function in attenuating coronary artery ligation induced cardiac hypertrophy and fibrosis. The extenuation of cardiac hypertrophy and fibrosis are significantly and positively correlated with the downregulated activities and gene expression of RAAS, ET-1, TNF-*α*, TGF-*β*_1_, and Hyp and increased concentration of NO. To the best of our knowledge, this is the first report to indicate JJYNJ negatively regulates cardiac hypertrophy and fibrosis in animal models, possibly through controlling the expression of neuroendocrine factors in the infarcted myocardium. Further investigations into the intracellular signaling pathways related to the antiremodeling effect of JJYNJ are ongoing.

## Figures and Tables

**Table 1:** Changes in hemodynamic parameters 4 weeks after coronary artery ligation (*n* = 10 in each group).

	Sham	CAL	JJYNJ
HR (*n*/min)	480 ± 33	488 ± 16	501 ± 19
SBP (mmHg)	149.73 ± 7.26	139.38 ± 11.98^*∗*^	159.66 ± 15.09^#^
DBP (mmHg)	113.13 ± 6.23	119.30 ± 13.24^*∗*^	113.26 ± 7.27^#^
PP (mmHg)	36.60 ± 7.17	24.42 ± 11.28^*∗*^	26.40 ± 9.77^§^
MABP (mmHg)	128.31 ± 6.11	131.95 ± 11.33	146.57 ± 10.46^#§^
LVSP (mmHg)	164.00 ± 16.00	127.70 ± 31.20^*∗*^	140.68 ± 20.17^§^
LVEDP (mmHg)	−6.64 ± 6.86	29.64 ± 10.67^*∗*^	14.94 ± 6.00^#§^
+*dp*/*dt*_max_ (mmHg/s)	11605.58 ± 1297.74	10537.06 ± 2611.57	9837.45 ± 859.36^§^
−*dp*/*dt*_max_ (mmHg/s)	11195.70 ± 1124.42	9815.84 ± 1429.14^*∗*^	9842.38 ± 973.28^§^

Values are expressed as means ± standard deviation; ^*∗*^ *p* < 0.05 when compared to the sham; ^#^ *p* < 0.05 when compared to the CAL; ^§^ *p* < 0.05 when compared to the sham.

**Table 2:** Changes in left ventricular weight, body weight, heart weight, LVW/BW, and HW/BW at 4 weeks after coronary artery ligation (*n* = 10 in each group).

	Sham	CAL	JJYNJ
LVW (mg)	811.63 ± 50.66	837.80 ± 77.74	869.10 ± 62.56
HW (mg)	1048.50 ± 52.62	1098.30 ± 99.68	1114.90 ± 83.56
BW (g)	362.50 ± 19.82	349.00 ± 25.03	374.50 ± 23.15^#^
LVW/BW (mg/g)	2.24 ± 0.09	2.40 ± 0.16^*∗*^	2.32 ± 0.14
HW/BW (mg/g)	2.89 ± 0.12	3.15 ± 0.20^*∗*^	2.98 ± 0.19^#^

Values are expressed as means ± standard deviation; ^*∗*^ *p* < 0.05 when compared to the sham; ^#^ *p* < 0.05 when compared to the CAL.

## References

[1] Katz A. M. (2000). *Heart Failure: Pathophysiology, Molecular Biology, and Clinical Management*.

[2] Gao Y., Gao J. P., Chen C. X., Wang H. L., Guo J., Wu R. (2014). Beneficial effects of houttuynin on ventricular remodeling induced by coronary artery ligation in rats. *European Journal of Pharmacology*.

[3] Triposkiadis F., Karayannis G., Giamouzis G., Skoularigis J., Louridas G., Butler J. (2009). The sympathetic nervous system in heart failure. physiology, pathophysiology, and clinical implications. *Journal of the American College of Cardiology*.

[4] Yuan D., Sunouchi H., Sakurai T., Saito K., Kano Y. (2002). Pharmacological properties of traditional medicines (XXVII). Interaction between Ephedra Herb and Gypsum under hyperthermal conditions in rats. *Biological and Pharmaceutical Bulletin*.

[5] Oh J. K., Hyun S. Y., Oh H. R. (2007). Effects of *Anemarrhena asphodeloides* on focal ischemic brain injury induced by middle cerebral artery occlusion in rats. *Biological and Pharmaceutical Bulletin*.

[6] Xiao S.-Z., Xu M.-E., Ge Y.-K., Xiao G.-F. (2006). Inhibitory effects of saponins from *Anemarrhena asphodeloides* Bunge on the growth of vascular smooth muscle cells. *Biomedical and Environmental Sciences*.

[7] Gu W.-L., Chen C.-X., Wu Q., Lü J., Liu Y., Zhang S.-J. (2010). Effects of Chinese herb medicine Radix Scrophulariae on ventricular remodeling. *Pharmazie*.

[8] Huang X. Y., Chen C. X., Zhang X. M., Liu Y., Wu X. M., Li Y. M. (2012). Effects of ethanolic extract from Radix Scrophulariae on ventricular remodeling in rats. *Phytomedicine*.

[9] Li Y.-S., Chen Z.-J., Zhu D.-Y. (2005). A novel bis-furan derivative, two new natural furan derivatives from *Rehmannia glutinosa* and their bioactivity. *Natural Product Research*.

[10] Kim S.-S., Son Y.-O., Chun J.-C. (2005). Antioxidant property of an active component purified from the leaves of paraquat-tolerant *Rehmannia glutinosa*. *Redox Report*.

[11] Zhou J., Xu G., Yan J. (2015). *Rehmannia glutinosa* (Gaertn.) DC. polysaccharide ameliorates hyperglycemia, hyperlipemia and vascular inflammation in streptozotocin-induced diabetic mice. *Journal of Ethnopharmacology*.

[12] Kou J., Yu B., Xu Q. (2005). Inhibitory effects of ethanol extract from Radix Ophiopogon japonicus on venous thrombosis linked with its endothelium-protective and anti-adhesive activities. *Vascular Pharmacology*.

[13] Kou J., Tian Y., Tang Y., Yan J., Yu B. (2006). Antithrombotic activities of aqueous extract from *Radix Ophiopogon japonicus* and its two constituents. *Biological and Pharmaceutical Bulletin*.

[14] Trindade D. C., Trindade R. C., Marassi M. P. (2007). Role of renin-angiotensin system in development of heart failure induced by myocardial infarction in rats. *Anais da Academia Brasileira de Ciencias*.

[15] Shimoyama M., Hayashi D., Takimoto E. (1999). Calcineurin plays a critical role in pressure overload-induced cardiac hypertrophy. *Circulation*.

[16] Zhou X., Gan P., Hao L. (2014). Antiinflammatory effects of orientin-2"-O-galactopyranoside on lipopolysaccharide-stimulated microglia. *Biological and Pharmaceutical Bulletin*.

[17] Shinzato T., Ohya Y., Nakamoto M., Ishida A., Takishita S. (2007). Beneficial effects of pioglitazone on left ventricular hypertrophy in genetically hypertensive rats. *Hypertension Research*.

[18] Livak K. J., Schmittgen T. D. (2001). Analysis of relative gene expression data using real-time quantitative PCR and the 2^−ΔΔ*C*_*T*_^ method. *Methods*.

[19] Tiyyagura S. R., Pinney S. P. (2006). Left ventricular remodeling after myocardial infarction: past, present, and future. *Mount Sinai Journal of Medicine*.

[20] Delcayre C., Silvestre J. S., Garnier A. (2000). Cardiac aldosterone production and ventricular remodeling. *Kidney International*.

[21] Hsueh W. A., Do Y. S., Jeyaseelan R. (1998). Angiotensin II and cardiac remodeling. *Mount Sinai Journal of Medicine*.

[22] Pellieux C., Foletti A., Peduto G. (2001). Dilated cardiomyopathy and impaired cardiac hypertrophic response to angiotensin II in mice lacking FGF-2. *Journal of Clinical Investigation*.

[23] Brilla C. G., Zhou G., Matsubara L., Weber K. T. (1994). Collagen metabolism in cultured adult rat cardiac fibroblasts: response to angiotensin II and aldosterone. *Journal of Molecular and Cellular Cardiology*.

[24] Takano H., Hasegawa H., Nagai T., Komuro I. (2003). Implication of cardiac remodeling in heart failure: mechanisms and therapeutic strategies. *Internal Medicine*.

[25] Watanabe T., Barker T. A., Berk B. C. (2005). Angiotensin II and the endothelium: diverse signals and effects. *Hypertension*.

[26] Bozkurt B., Kribbs S. B., Clubb F. J. (1998). Pathophysiologically relevant concentrations of tumor necrosis factor-*α* promote progressive left ventricular dysfunction and remodeling in rats. *Circulation*.

[27] Fu Y.-C., Chi C.-S., Yin S.-C., Hwang B., Chiu Y.-T., Hsu S.-L. (2004). Norepinephrine induces apoptosis in neonatal rat cardiomyocytes through a reactive oxygen species-TNF*α*-caspase signaling pathway. *Cardiovascular Research*.

[28] Gurantz D., Cowling R. T., Villarreal F. J., Greenberg B. H. (1999). Tumor necrosis factor-*α* upregulates angiotensin II type 1 receptors on cardiac fibroblasts. *Circulation Research*.

[29] Bujak M., Frangogiannis N. G. (2007). The role of TGF-*β* signaling in myocardial infarction and cardiac remodeling. *Cardiovascular Research*.

[30] Rosenkranz S. (2004). TGF-*β*1 and angiotensin networking in cardiac remodeling. *Cardiovascular Research*.

[31] Ikeuchi M., Tsutsui H., Shiomi T. (2004). Inhibition of TGF-*β* signaling exacerbates early cardiac dysfunction but prevents late remodeling after infarction. *Cardiovascular Research*.

[32] Diniz G. P., Carneiro-Ramos M. S., Barreto-Chaves M. L. M. (2007). Angiotensin type 1 (AT1) and type 2 (AT2) receptors mediate the increase in TGF-*β*1 in thyroid hormone-induced cardiac hypertrophy. *Pflugers Archiv European Journal of Physiology*.

[33] Gray M. O., Long C. S., Kalinyak J. E., Li H.-T., Karliner J. S. (1998). Angiotensin II stimulates cardiac myocyte hypertrophy via paracrine release of TGF-*β*
_1_ and endothelin-1 from fibroblasts. *Cardiovascular Research*.

[34] Schneider M. D. (2002). Serial killer: angiotensin drives cardiac hypertrophy via TGF-*β*1. *The Journal of Clinical Investigation*.

[35] Zhao X.-S., Pan W., Bekeredjian R., Shohet R. V. (2006). Endogenous endothelin-1 is required for cardiomyocyte survival in vivo. *Circulation*.

[36] Nguyen Q. T., Cernacek P., Calderoni A. (1998). Endothelin A receptor blockade causes adverse left ventricular remodeling but improves pulmonary artery pressure after infarction in the rat. *Circulation*.

[37] Iwanaga Y., Kihara Y., Hasegawa K. (1998). Cardiac endothelin-1 plays a critical role in the functional deterioration of left ventricles during the transition from compensatory hypertrophy to congestive heart failure in salt-sensitive hypertensive rats. *Circulation*.

[38] Lerman A., Hildebrand F. L., Aarhus L. L., Burnett J. C. (1991). Endothelin has biological actions at pathophysiological concentrations. *Circulation*.

[39] Ruiz-Stewart I., Tiyyagura S. R., Lin J. E. (2004). Guanylyl cyclase is an ATP sensor coupling nitric oxide signaling to cell metabolism. *Proceedings of the National Academy of Sciences of the United States of America*.

[40] Tiyyagura S. R., Kazerounian S., Schulz S., Waldman S. A., Pitari G. M. (2004). Reciprocal regulation and integration of signaling by intracellular calcium and cyclic GMP. *Vitamins & Hormones*.

[41] Cheng T.-H., Shih N.-L., Chen S.-Y. (2005). Nitric oxide inhibits endothelin-1-induced cardiomyocyte hypertrophy through cGMP-mediated suppression of extracellular-signal regulated kinase phosphorylation. *Molecular Pharmacology*.

[42] Miyamoto T., Takeishi Y., Shishido T. (2003). Role of nitric oxide in the progression of cardiovascular remodeling induced by carotid arterio-venous shunt in rabbits. *Japanese Heart Journal*.

[43] Weber K. T., Brilla C. G. (1993). Structural basis for pathologic left ventricular hypertrophy. *Clinical Cardiology*.

[44] Makino T., Ito Y., Sasaki S.-Y., Fujimura Y., Kano Y. (2004). Preventive and curative effects of *Gyokuheifu-san*, a formula of traditional Chinese medicine, on allergic rhinitis induced with Japanese cedar pollens in guinea pig. *Biological and Pharmaceutical Bulletin*.

